# New developments in the pathology of malignant lymphoma: a review of the literature published from January to April 2017

**DOI:** 10.1007/s12308-017-0295-x

**Published:** 2017-07-22

**Authors:** J. Han van Krieken

**Affiliations:** Department of Pathology, Radboud University Medical Centre, P.O. Box 9101, 6500 HB Nijmegen, The Netherlands

## Introduction

This issue’s review contains, like always, a nice series of new data that hopefully satisfies the interest of the reader. No real theme, but a host of different developments.

## Biology of lymphoma

As is well known, the process of antigen receptor rearrangements can lead to mistakes resulting in oncogenic chromosomal translocations. The enzyme activation-induced cytidine deaminase (AID) plays a role in the initiation of class switching in B-lymphocytes, and therefore, its expression is tightly controlled by the phosphatidylinositol 3-kinase δ (PI3Kδ) pathway. Compagno et al. [1] show that treatment of primary mouse B cells with idelalisib or duvelisib, and to a lesser extent ibrutinib, enhances the expression of AID resulting in increased frequency of somatic hypermutation and chromosomal translocation, not only of the IgH locus but also to several other sites in the genome. Furthermore, in mice, PI3Kδ inhibitors or ibrutinib increases the formation of AID-dependent tumors. Consistently, PI3Kδ inhibitors enhanced AID expression and translocation frequency to IGH and other sites in human chronic lymphocytic leukemia (CLL) and mantle cell lymphoma (MCL) cell lines, and, finally, in patients treated with idelalisib, but not ibrutinib, there is increased somatic hypermutation in the genome. This worrisome effect should be carefully monitored in patients treated with these agents, especially since patients are often treated for a long time and the effects maybe be seen only after quite some time.

Classification (and understanding!) of tumors is based on the cell of origin concept. Very distinct malignancies like classical Hodgkin lymphoma (cHL), CLL, and multiple myeloma (MM) arise from B lymphocytes, but have various stages of differentiation. Law et al. [2] performed a large-scale genome-wide association study in many patients (CLL: *n* = 1842; HL: *n* = 1465; MM: *n* = 3790) to see whether there might be a common predisposition for B cell lineage-derived neoplasia. They identified a risk locus at 3q22.2 with opposing effects between CLL and cHL. Eight established non-HLA risk loci showed also pleiotropic associations. Within the HLA region, Ser37 + Phe37 in HLA-DRB1 was associated with increased CLL and cHL risk and reduced MM risk, and Gly70 in HLA-DQB1 showed opposing effects between CLL and cHL. These data identify shared biological pathways influencing the development of CLL, HL, and MM but with opposing risks and thus maybe are active at various stages of B cell development.

### Hodgkin lymphoma

HL remains an enigmatic disease with so many different aberrant processes involved. Zahn et al. [3] add a new mechanism, namely the presence of a truncated form of PTPN1 in cHL cells (both in cell lines and in tissues), which upregulates Janus kinase/signal transducer and activator of transcription (JAK/STAT) activity, a common feature of B cell lymphomas. Functional characterization of this variant further indicated that it leads to proliferation and reduced sensitivity to chemotherapy. This latter finding is a bit surprising, cHL being one of the earliest and well-known diseases curable by chemotherapy. Another new mechanism in HL is described by Agostinelli et al. [4]. They analyzed the functional status of glycogen synthase kinase-3 beta (GSK-3β) in cHL using antibodies raised against fixation-resistant epitopes of phospho Y216 GSK-3β (active form), phospho S9 GSK-3β (inactive form), and β-catenin protein. GSK-3β is a serine/threonine kinase involved in glycogen metabolism, cell cycle progression, differentiation, embryogenesis, migration, metabolism, survival, and cellular senescence. Its main biological function is to inhibit β-catenin by sequestration, and promotion of its proteasomal degradation in the Wnt canonical pathway. The active form of the enzyme was detected in 100 of 100 (100%) of the cases, and in line with that, the β-catenin protein was expressed in only 12 of 100 (12%) of the samples. These findings imply that activation of GSK-3β in cHL results in inhibition of the Wnt/β-catenin signal cascade and the aberrant accumulation of its activated form in nuclei of Reed-Sternberg and Hodgkin cells. A third new mechanism is described by Etzel et al. [5]. Because it is known that survival and proliferation of Hodgkin and Reed-Sternberg (HRS) cells are dependent on constitutive activation of nuclear factor κB (NF-κB) they looked for mutations in TNF alpha-induced protein 3 (TNFAIP_3_), one of the inhibitors of NF-κB. In addition, they evaluated protein expression by immunohistochemistry. Of the 37 investigated cHL cases, 8 (22%) had a mutation and these tended to be negative by immunohistochemistry. All these cases were negative for EBV which suggests complementing functions of TNFAIP_3_ inactivation and EBV infection in cHL pathogenesis.

The same gene was studied by Jung et al. [6] in ocular marginal zone lymphoma (oMZL). They analyzed 10 cases using whole-genome and RNA sequencing and an additional 38 cases with targeted sequencing. Major genetic alterations affecting genes involved in NF-κB pathway activation (60%), chromatin modification and transcriptional regulation (44%), and B cell differentiation (23%) were identified. In whole-genome sequencing, the 6q23.3 region containing TNFAIP_3_ was deleted in five samples (50%). In addition, five structural variation breakpoints in the first intron of IL20RA located in the 6q23.3 region were found in three samples (30%). In targeted sequencing, a disruptive mutation of TNFAIP_3_ was the most common alteration (54%), followed by mutations of TBL1XR1 (18%), CREBBP (17%), and KMT2D (6%). All TBL1XR1 mutations were located within the WD40 domain, and TBL1XR1 mutants transfected into 293 T cells increased TBL1XR1 binding with nuclear receptor corepressor (NCoR), leading to increased degradation of NCoR and the activation of NF-κB and JUN target genes. This study confirms that genes involved in the activation of the NF-κB signaling pathway are the major driver in the oncogenesis of ocular MZL, which is inline findings in MZL at other sites, especially nodal based ones [7], which are, as earlier described in this Journal, difficult to separate from follicular lymphoma (FL) [8].

### B cell lymphomas

Although the primary genetic alteration in FL, the t(14;18), is among the earliest detected tumor-associated translocations, the full pathogenesis is not clear. Especially, the importance of the tumor stroma is recognized but not understood. Pandey et al. [9] demonstrate that FL stromal cells overexpress CXCL12. Interleukin-4 (IL-4) high FL-T_FH_ cells, unlike FL B cells themselves, trigger CXCL12 upregulation in human stromal cell precursors. In agreement, they found that expression of CXCL12 is associated with IL-4 expression and signaling within the FL stroma. In an in vitro model, they confirmed that the IL-4/CXCL12 axis was amplified in activated lymphoid stromal cells. Finally, CXCL12 triggers primary FL B cell activation, migration, and adhesion, a process antagonized by BTK and PI3K inhibitors. These data identify the IL-4/CXCL12 loop as a previously unrecognized pathway involved in FL stroma and as a potential therapeutic target in FL patients.

It is well known that activated B cell-like (ABC) and germinal center B cell-like (GCB) diffuse large B cell lymphoma (DLBCL) represent the two major molecular DLBCL subtypes. They are characterized by differences in clinical course and by divergent addiction to oncogenic pathways. Erdmann et al. [10] determined activity of novel compounds in these two subtypes by conducting an unbiased pharmacologic in vitro screen. The PI3K alpha/delta (PI3Kα/δ) inhibitor AZD8835 showed marked potency in ABC DLBCL cells, whereas the protein kinase B (AKT) inhibitor AZD5363 induced apoptosis in PTEN-deficient DLBCLs irrespective of the molecular subtype. These in vitro results were confirmed in various cell line xenograft and patient-derived xenograft mouse models in vivo. Treatment with AZD8835 induced inhibition of NF-κB signaling, prompting us to combine AZD8835 with the Bruton’s tyrosine kinase inhibitor ibrutinib. This combination was synergistic and effective both in vitro and in vivo. In contrast, the AKT inhibitor AZD5363 was effective in PTEN-deficient DLBCLs through downregulation of the oncogenic transcription factor MYC. These data suggest that patients should be stratified according to their oncogenic dependencies when treated with PI3K and AKT inhibitors. Of course, this needs conformation in the clinical setting.

Components of the B cell receptor (BCR) signaling pathway represent promising therapeutic targets in DLBCL and other B cell malignancies. MYC, a transcriptional factor and oncoprotein, is overexpressed in a proportion of DLBCL and indicates poor prognosis and aggressive clinical course. Wang et al. [11] tried to elucidate the BCR signaling pathway in MYC-positive DLBCL by analyzing the levels of BCR-associated genes according to MYC gene status and outcome. They found that CD19, SYK, and BLK were highly expressed in DLBCL with MYC gene overexpression. MYC-positive DLBCL had higher levels of pSYK and pBLK, but only pSYK level correlated with patient survival. In vitro studies demonstrated that overexpression of the MYC gene augmented BCR signaling, whereas MYC gene knockdown attenuated BCR signaling. Thus, MYC protein-positive DLBCL features highly activated BCR signaling and may represent a potential candidate for BCR inhibitor therapy.

### T cell lymphoma

Refractory coeliac disease (rCD) is a problematic condition in which about 50% of cases develop into an aggressive incurable T cell lymphoma, enteropathy-associated T cell lymphoma (EATL). Although rCD was recently redefined according to the WHO classification, the present literature still uses the distinction into two types, rCD I and rCDII. Ritter et al. [12] performed high-throughput sequencing of the TCRβ rearrangements and compared the results from duodenal mucosa specimens of controls (*n* = 9), patients with active CD (*n* = 10), CD patients on a gluten-free diet (*n* = 9), rCD type I (*n* = 8), rCD type II (*n* = 8), and unclassified Marsh I cases (*n* = 3) collected from 2002 to 2013. On average, 10^6^ sequence reads per sample were generated consisting of up to 900 individual TCRβ rearrangements. In rCD type II, the most frequent clonotypes (i.e., sequence reads with identical CDR3) represent in average 43% of all, which was significantly higher than that in controls (6.8%) or rCD type I (6.7%). Repeat endoscopies in individual patients revealed stability of clonotypes for up to several years without clinical symptoms of EATL. Dominant clonotypes identified in individual patients with rCD type II were unique and not related between patients. CD-associated, gliadin-dependent CDR3 motifs were only detectable at low frequencies. These results support the assumption that the clonal T cells expand independent of gluten stimulation. This work adds significantly to the knowledge on this disease, which had been widely investigated. Nevertheless, it is a very rare condition according to van Wanrooij et al. [13]. They evaluated the 106 CD patients who were referred to their tertiary referral center between January 2006 and December 2011 for evaluation of non-responsive CD. In addition, a questionnaire was sent to all 82 gastroenterology departments in the Netherlands to reveal whether a patient with rCD was currently being evaluated or had been treated between 2006 and 2012. During this 6-year period, a total of 31 patients were diagnosed with rCD (19 rCD I and 12 rCD II). The nationwide survey revealed five additional patients with rCD I and one patient with rCD II. This leads to an annual incidence of rCD of 0.83/10.000 CD patients. The remaining patients with non-responsive CD were diagnosed with involuntary gluten ingestion (21.7%), delayed mucosal recovery (11.3%), enteropathy-associated T cell lymphoma (7.5%), and autoimmune enteropathy (1.8%). This well-conducted nationwide study reveals a very low incidence of rCD in the Netherlands. Nevertheless, rCD is a clinically relevant disease entity in CD patients non-responsive to the gluten-free diet.

Angioimmunoblastic T cell lymphoma (AITL) originates from follicular helper T cells and is characterized by a polymorphic infiltrate with the neoplastic T cells forming small clusters around the follicle and high endothelial venules. Wang et al. [14] performed whole-exome sequencing in nine cases of AITL from Taiwan (*n* = 6) and U.K. (*n* = 3). They confirmed frequent mutations in TET2 (9/9), DNMT3A (3/9), IDH2 (3/9), RHOA (3/9), and PLCG1 (2/9). Furthermore, they identified mutations in TNFRSF21 (1/9), CCND3 (1/9), and SAMSN1 (1/9), which had not yet been implicated in the pathogenesis of AITL. McKinney et al. [15] performed a similar analysis in 68 cases of hepatosplenic T cell lymphoma (HSTL), a rare and lethal lymphoma. Chromatin-modifying genes, including *SETD2*, *INO80*, and *ARID1B*, were mutated in 62%; there were frequent mutations in *STAT5B* (31%), *STAT3* (9%), and *PIK3CD* (9%). In addition, they noted less frequent events in *EZH2*, *KRAS*, and *TP53*. *SETD2* was the most frequently silenced gene in HSTL. For some of these genetic events, targeted treatments have been described in other tumors and thus, these might be also effective in AITL.

## Epidemiology of lymphoma

Prevention of cancer is potentially a very important tool in decreasing its toll. The incidence of several forms of cancer is influenced by life style factors including smoking and diet. Han et al. [16] reviewed 221 published cohort and case-control studies that reported relative risk (RR) of non-Hodgkin lymphoma (NHL) and fat intake using PubMed, Cochrane, EMBASE, and Google Scholar databases. Only 10 of 221 studies (two cohort and eight case-control studies) were relevant to this meta-analysis. There was a significant association between total fat consumption and increased risk of NHL (RR = 1.26); in addition, subgroup analysis showed a significant correlation with DLBCL (RR = 1.41) but not with FL (RR = 1.21), CLL (RR = 0.91), nor with T cell lymphoma (RR = 1.12). These findings further indicate that different lymphoma types are really different diseases. However, reducing fat intake has probably more effect on other diseases than on the incidence of DLBCL. Kleinstern et al. [17] performed an interesting study on lifestyle risk factors for B-NHL. In a case-control study, they investigated self-reported medical and lifestyle exposures, for overall B-NHL and subtypes among Palestinian Arabs (PA) and Israeli Jews (IJ). They recruited 823 cases and 808 healthy controls. Among 307 PA/516 IJ, B-NHL cases subtype distributions differed, with DLBCL being prominent among PA (71%) compared to IJ (41%); FL was observed in 14 versus 28%, and MZL, in 2 versus 14%, respectively. Overall B-NHL in both populations was associated with recreational sun exposure (RR = 1.43), black hair-dye use (RR = 1.70), hospitalization for infection (RR = 1.68), and first-degree relative with hematopoietic cancer (RR = 1.69). An inverse association was noted with alcohol use (RR = 0.46). Subtype-specific exposures included smoking (FL, RR = 1.46) and more of ten monthly indoor pesticide use (DLBCL, RR = 2.01). Associations observed for overall B-NHL in PA only, included gardening (RR = 1.93) and history of herpes, mononucleosis, rubella, and blood transfusion (RR > 2.5), while for IJ, risk factors included growing fruits and vegetables (RR = 1.87) and self-reported autoimmune diseases (RR = 1.99). It is quite interesting that they found in two geographically proximate populations some unique risk factors for B-NHL. Heterogeneity in the observed associations by ethnicity could reflect differences in lifestyle, medical systems, and reporting patterns, while, like in the previous study, variations by histology infer specific etiologic factors for lymphoma subtypes.

Dhakal et al. [18] evaluated the effect of radiotherapy (RX) for ocular adnexal lymphoproliferative disorders (OALDs) in 112 patients, 71 MZL and 41 other types of lymphoma. Fifty-six patients with MZL OALD and 26 patients with non-MZL received RX only. Complete remission was achieved in 49 (87.5%) patients in the MZL cohort and 23 (88.4%) in the non-MZL cohort. Local recurrence occurred in four (7.1%) patients in the MZL cohort, compared with four (15.3%) patients in the non-MZL cohort. The 5-year and 10-year disease-free survival was 95 and 83% for the oMZL and 67 and 56% for the non-MZL. On multivariate analyses, age, non-MZL histology, and radiation dose <30 Gray were associated with worse survival. These results indicate a high cure rate with local therapy only.

Svendsen et al. [19] provide a good overview of their experience with the rare eyelid lymphoma, a nice example of international collaboration. Data from 86 patients were collected from 7 international eye cancer centers from1980–2015, indicating on average only one patient in 3 years! The cases included primary and secondary lymphomas affecting the eyelid. Mean age was 63 years and 47 (55%) were male. B cell lymphomas constituted 83% (*n* = 71) and T cell lymphomas constituted 17% (*n* = 15). The most common subtypes were MZL (37% [*n* = 32]), FL (23% [*n* = 20]), DLBCL (10% [*n* = 9]), MCL (8% [*n* = 7]), and mycosis fungoides (MF) (9% [*n* = 8]). EMZL had a female predilection (69% [22 of 32]), whereas MCL (71% [5 of 7]) and MF (88% [7 of 8]) had a male predominance. MCL (57% [4 of 7]), DLBCL (56% [5 of 9]), and MF (88% [7 of 8]) were frequently secondary involvement. Localized EMZL and FL were mostly treated with external beam radiation therapy, whereas DLBCL, MCL, and high Ann Arbor stage EMZL and FL were frequently treated with chemotherapy. DLBCL and MCL had a poor prognosis, whereas EMZL, FL, and MF had a good prognosis.

More common is primary central nervous system lymphomas (PCNSL), still a rare lymphoma, with a poor prognosis. Recently, an increased incidence has been reported. The present study is a population-based study of all patients with PCNSL in the Uppsala/Örebro region of middle Sweden on 96 patients (50 female) with a mean age of 66 (range 17–95). Enblad et al. [20] confirmed a statistically significant increase in age-standardized incidence. No patient had an HIV infection. Two patients had undergone kidney transplantation and were treated with immunosuppressive drugs. A high proportion of the patients, 29%, had a history of an autoimmune or inflammatory disease. The prognosis was poor with a median survival of only 4 months. In the 70 (73%) patients treated with curative intention, the median survival was 12 months. Patients treated with high-dose methotrexate, radiotherapy, and/or temozolomide appeared to have a better survival. There was no improvement in survival during the study period or after the introduction of rituximab. There also was no difference in any of the analyzed variables that could explain the increased incidence.

## Defining entities

### B cell lymphomas

De Souza et al. [21] found IgG4 expression in 4 out of 30 patients (13%) with primary cutaneous (PC) MZL. Patients with IgG4-positive lymphomas were 57 to 77 years of age (mean, 69) at biopsy. The lesions were solitary in two patients and were most commonly located on the trunk. Patients with IgG4-negative lymphomas experienced earlier disease onset at an average age of 53 years. The majority of the IgG4-negative cases presented with localized disease, on the trunk and upper extremities. There was no significant difference in the IgG4-positive versus IgG4-negative cases for the following parameters: Ig κ or λ restriction, B cell or T cell predominance, and site of the lesions. Detection of IgG4 expression is therefore not of diagnostic or clinical relevance.

Cao et al. [22] investigated MYD88 and CXCR4 mutations in various IgM macroglobulinemia-associated diseases, including Waldenstrom macroglobulinemia (WM), various types of B cell non-Hodgkin’s lymphoma (NHL), MM, primary amyloidosis (AL), and monoclonal gammopathy of undetermined significance (MGUS): IgM monoclonal gammopathy-related diseases (IgM-RD). Of their cohort of 112 patients, 64 (57%) carried the MYD88 L265P mutation and 14 patients (13%) carried the CXCR4 WHIM-like mutation; MYD88 L265P in cases with WM (39/42), MGUS (8/18), NHL (14/41, including 4/13 DLBCL, 1/8 eMZL, 3/6 (sMZL), 1/4 chronic lymphocytic leukemia, 2/3 nodal (n)MZL, 1/2 MCL, 1 BL, and 1 B cell NHL that could not be classified), primary AL (2/2), and IgM-PN (1/1). The mutation was absent in five patients with cryoglobulinemia, two with primary cold agglutinin disease and one with MM. The CXCR4 WHIM-like mutation was present in 10/42 patients with WM, 3/41 with NHL (1 DLBCL, 1 SMZL, and 1 NMZL), and 1/18 patients with IgM MGUS. Among the patients with NHL, those with the mutated MYD88 L265P genotype were younger and had lower level of IgG and IgA than the patients with the wild-type genotype. The authors suggest that MYD88 mutation status might be used to differentiate among diseases, but in fact, their data do not give much hope for that.

Rohde et al. [23] found in pediatric BL a mutation frequency of 78% for *ID3*, 13% for *TCF3*, and 36% for *CCND3*. *ID3* and *CCND3* mutations were associated with more advanced stages of the disease and thus may represent a highly relevant second hit in pediatric BL pathogenesis (adult cases were not investigated).

Colomo et al. [24] investigated BL as well, but also cases of DLBCL with MYC and BCL2/6 translocations to see whether they could prescreen cases with immunohistochemistry to avoid mutation testing in many DLBCL cases without MYC translocation. LMO2 is a germinal center marker expressed in most lymphomas originated in these cells. Because in gene expression profiling studies, LMO2 downregulation is observed in BL and DLBCL with MYC translocations, they analyzed LMO2 protein expression in 46 BLs and 284 BLBCL. All 46 BL lymphomas had a MYC rearrangement and LMO2 was expressed in 1 (2%). LMO2 was also positive in 146/268 (55%) DLBCL cases, but only in 6/42 (14%) DLBCL with MYC translocation and in 2/16 (12.5%) double hit lymphomas. The relationship between LMO2 negativity and MYC translocation was further analyzed in different subsets of tumors according to CD10 expression and cell of origin. Lack of LMO2 expression was associated with the detection of MYC translocations with high sensitivity (87%), specificity (87%), positive predictive value and negative predictive value (74 and 94%, respectively), and accuracy (87%) in CD10 DLBCL. This indicates that LMO2 surpassed MYC in CD10 DLBCL as predictor of MYC status. Although the authors conclude that their findings suggest that LMO2 loss may be a good predictor for the presence of MYC translocation in CD10 DLBCL, I believe that still too many cases are missed whilst the benefit of avoiding mutation testing is only limited.

Bogisz et al. [25] are also interested in DLBCL with MYC and BCL2, but focus on protein expression and found that in cases with high expression of both, there is also high BCR-mediated signaling using their previously identified signature of active BCR signaling on formalin-fixed paraffin-embedded specimens. Their findings suggest that the BCR signaling pathway may be more active in MYC/BCL2 double-expressor DLBCL and thus may represent a rational therapeutic target in this aggressive DLBCL subgroup.

### T cell lymphomas

According to Wang et al. [26]. it is worthwhile to prescreen anaplastic large cell lymphomas (ALCLs) with p63 immunohistochemistry to select for cases with chromosomal rearrangements of the TP53 homolog TP63, an indicator of aggressive clinical behavior. Of their 116 ALCLs, 35% were positive for p63 protein. Primary cutaneous and anaplastic lymphoma kinase (ALK)-negative ALCLs were more frequently positive than ALK-positive ALCLs. Cases with TP63 gene rearrangements showed uniform p63 expression. In nonrearranged cases, p63 expression was associated with extra copies of TP63 on 3q28, which correlated with extra copies of the DUSP22 locus on 6p25.3. Results of immunohistochemistry, Western blotting, and RNA sequencing indicated that p63 expression in nonrearranged cases was entirely attributable to TAp63 isoforms. These findings indicate that ALCLs without TP63 rearrangements may express TAp63 isoforms of p63 and that this expression is associated with extra copies of TP63, probably due to widespread genomic copy number abnormalities rather than focal gains. Immunohistochemistry for p63 in ALCL is not specific for TP63 rearrangements but is useful as a screening test to select cases for further testing by fluorescence in situ hybridization (FISH). Immunohistochemistry for ΔNp63 (p40) is not informative in the evaluation of ALCL.

Inhibition of the Janus kinase (JAK)-STAT pathway has been implicated as a treatment option for extranodal natural killer/T cell lymphoma, nasal type (NTCL) but a predictive marker is not yet available. Sim et al. [27] found in 5 of 71 patients with NTCL (7%) JAK3 mutations in the pseudokinase domain. Proliferation of Ba/F3 cells transduced with these novel JAK3 mutations was independent of IL-3 and was inhibited by the JAK3 inhibitor tofacitinib. Although phosphorylated STAT3 was overexpressed in 35 of 68 patients with NTCL (51%), a STAT3 mutation (p.Tyr640Phe; STAT3^Y640F^) at the SRC homology 2 domain was detected in only 1 of the 63 patients (<2%). A STAT3 inhibitor was active against STAT3-mutant SNK-6 and YT cells. These results indicate that novel JAK3 mutations are oncogenic and druggable in NTCL. The authors conclude that the JAK3 or STAT3 signal was altered in NTCL, and pathway inhibition might be a therapeutic option for patients with JAK3- or STAT3-mutant NTCL.

## New entities/subtypes

We are less than a year after the presentation of the updated, but not new, WHO classification, but there is already quite some new data that show once again that a classification is always work in progress.

It is well known that EBV can give rise to a large variation of neoplasms, including a spectrum of lymphoproliferations in various forms of immunodeficiency. Human herpesvirus 8 (HHV8)-associated lymphoid proliferations are way less common and poorly characterized disorders mainly affecting immunosuppressed patients, especially with HIV infection. Now, based on 66 biopsies from 61 patients (47 HIV-positive), Gonzalez-Farre et al. [28] describe the clinicopathological spectrum of these lesions. The series consisted of 13 (20%) cases of HHV8-related reactive lymphoid hyperplasia, 2 (3%) HHV8 plasmablastic proliferations of the splenic red pulp, 28 (42%) multicentric Castleman disease, 6 (9%) germinotropic lymphoproliferative disorders, and 17 (26%) HHV8-related lymphomas. As expected, the pathologic diagnosis was predictive of overall survival. In addition to the classical presentation of the different entities, they identified novel and overlapping features. Reactive HHV8 proliferations were frequently associated with systemic symptoms but never progressed to overt HHV8-positive lymphoma. Two cases had a plasmablastic proliferation limited to spleen. Eight cases of multicentric Castleman disease had a previously unrecognized presentation shortly after the diagnosis of HIV infection, six cases had cavity effusions, and three showed plasmablast-enriched proliferations. One germinotropic lymphoproliferative disorder was EBV-negative and three occurred in HIV-positive patients, who had distinctive clinical and morphological features. Two of the HHV8-related lymphomas did not fulfill the criteria for previously recognized entities. All these findings expand the clinical and pathological spectrum of HHV8-related lymphoid proliferations, which is broader than currently recognized.

Boyer et al. [29] describe an interesting group of lesions, based on their experience with 12 cases and review of the literature. Previously, case reports presented localized fibrin-associated Epstein-Barr virus (EBV)+ large B cell proliferations at unusual anatomic sites. These have been included in the category of DLBCL associated with chronic inflammation (DLBCL-CI) in the WHO classification. Median age was 55 years with a male:female ratio of 3. In their 12 cases, the lymphoma was an incidental microscopic finding involving atrial myxomas (*n* = 3), thrombi associated with endovascular grafts (*n* = 3), chronic hematomas (*n* = 2), and pseudocysts (*n* = 4). All cases tested were nongerminal center B cell origin, type III EBV latency, and were negative for MYC rearrangements and alternative lengthening of telomeres by FISH. Most showed high CD30, Ki67, and PD-L1, and low to moderate MYC and p53 expression. Among 11 patients with detailed follow-up, 6 were treated surgically, 3 with cardiac or vascular lesions had persistent/recurrent disease at intravascular sites, and 4 died of causes not directly attributable to lymphoma. Reports of previously published fibrin-associated cases showed similar features, whereas traditional DLBCL-CI cases with a mass lesion had significantly higher lymphoma-associated mortality. Fibrin-associated EBV+ large B cell lymphoma is clinicopathologically distinct from DLBCL-CI, warranting separate classification. Most cases, particularly those associated with pseudocysts, behave indolently with the potential for cure by surgery alone and may represent a form of EBV+ lymphoproliferative disease rather than lymphoma. However, primary cardiac or vascular disease may have a higher risk of recurrence despite systemic chemotherapy. These interesting lesions might have a relation with EBV+ mucocutaneous ulcer.

Guitart et al. [30] provide further data from 34 patients with primary cutaneous CD8-positive aggressive epidermotropic T cell lymphoma, a rare and poorly characterized variant of cutaneous lymphoma, still considered a provisional entity in the latest 2016 WHO classification update. The median age was 77 years (range 19–89 years) and presentation was primarily with extensive annular necrotic plaques or tumor lesions with frequent mucous membrane involvement. The 5-year survival was 32% with a median survival of 12 months. A subset of 17 patients had a prodrome of chronic patches prior to the development of aggressive ulcerative lesions. There were cases with lack of CD8 or αβ T cell receptor expression yet with similar clinical and pathological presentation. The authors recommend the term primary cutaneous aggressive epidermotropic cytotoxic T cell lymphoma as this broader designation better describes this clinical-pathologic presentation, which allows the inclusion of cases with CD8-negative and/or αβ/γδ T cell receptor chain double-positive or double-negative expression.

Hu et al. [31] describe 12 patients with EBV-positive intestinal T/natural killer (NK) cell lymphoma (ITNKL), an uncommon tumor with an extremely aggressive clinical behavior. All patients had involvement of the small intestine with concurrent lesions of the large intestine in two. Seven patients (58%) had Lugano stages IIE/IV disease and eight (67%) were categorized as high-intermediate/high-risk according to the prognostic index for PTCL (PIT). Three patients (25%) with an age of onset of less than 50 years had chronic active EBV infection (CAEBV). Five CD56-positive patients (42%) had a poorer prognosis than those without CD56 expression. NK cell-type lymphoma defined by the absence of any TCR expression or clonal TCR-γ rearrangement was found in six patients (50%). Interestingly, EBV positive intra-epithelial lymphocytosis was observed in one case with a background of CAEBV.

## Pitfalls in lymphoma diagnosis

According to Tomasini et al. [32] chronic amastigote-negative cutaneous leishmaniasis (CL) might be misdiagnosed as lymphoma. It is a diagnostic challenge, as the parasite load may be low or absent in biopsy tissue sections. They analyzed a series of consecutive biopsy specimens, taken from 130 patients with a diagnosis of granulomatous dermatitis of unknown etiology. A total of 27 of 130 samples were positive for Leishmania-specific DNA. In only three patients, a clinical diagnosis CL was made. The lesions were single or multiple nodules or plaques of many months of duration. Histopathologically, a tuberculoid granulomatous dermatitis was the least common denominator in every case, whilst in five cases, a heavy lymphoid component was predominant. One patient had a concurrent cutaneous marginal zone lymphoma (MZL); the additional PCR study showed the presence of Leishmania DNA in tissue. The results of this study expand on previous observations as to the deceptive clinicopathologic manifestations of chronic CL, confirming the diagnostic value of PCR analysis for Leishmania DNA in unspecified granulomatous dermatitides. The authors also suggest that, in countries where leishmaniasis is endemic, PCR for Leishmania-specific DNA be performed in any idiopathic pseudolymphomatous. They also indicate that further study as to whether chronic Leishmania infection is implicated in the pathogenesis of some cutaneous MZL is warranted.

Eladl et al. [33] provide further data on the well-known pitfall of the presence of HRS-like B cells in peripheral T cell lymphoma (PTCL). They describe 30 such cases from Japan. Twenty-three cases (77%) were of follicular T-helper phenotype (TFH) derivation: 12 were AILT and 11 PTCL with TFH phenotype (PTCL-TFH). The remaining seven cases were diagnosed as PTCL, not otherwise specified (PTCL-NOS). EBV was detected in 25 cases (83%), but HRS-like B cells were EBER-positive in only 20 cases (67%). Most of the patients presented at an advanced clinical stage. The 3-year overall and progression-free survival rates were 44 and 27%, respectively. No significant clinicopathological differences were detected between PTCL-TFH, PTCL-NOS, and the angioimmunoblastic cases. Although the authors conclude that HRS-like B cells in a subset of T cell lymphomas were present more often in elderly patients and associated with advanced clinical stages and dismal prognosis, the finding is actually of little practical value.

## Prognostic factors in lymphoma

Only a few prognostic studies this time. Koh et al. [34] found with immunohistochemistry on biopsies from 125 patients with cHL GLUT1, PD-L1, PD-L2, and PD-1 expression in 45, 63, 10, and 14%, respectively. There was a positive correlation between GLUT1 and PD-L1 expression and between GLUT1 and PD-L2 expression. GLUT1 expression in HRS cells was not associated with overall survival or event-free survival although in the subgroup with high stage, it was associated with better event-free survival. Hong et al. [35] measured the pretreatment serum level of survivin in 210 DLBCL patients and analyzed its association with survival outcome and EBV status, as represented by EBV-encoded RNA (EBER) in DLBCL. Mean serum survivin level was higher in patients with relapsed or refractory disease than with responsive disease. Serum survivin-positive patients had worse overall and progression-free survival. Serum survivin positivity was associated with unfavorable characteristics including stage. In patients with non-GCB-cell-type DLBCL, serum survivin-positive patients also had significantly worse survival than serum survivin-negative patients. EBER-positivity was found in 7%, and EBER-positive patients had worse survival. Patients having concomitant positivity for serum survivin and EBER expression showed extremely poor prognosis. In the present era of rituximab, DLBCL with serum survivin positivity showed adverse clinical features and followed worse clinical course, especially in non-GCB subtype DLBCL. EBER-positivity was still associated with worse outcomes in DLBCL. Satou et al. [36] compared the clinicopathologic characteristics of 37 cases of MUM1-positive and 51 cases of MUM1-negative BL in Japan. Patients with MUM1-negative BL were significantly younger and were more often female. Among the BL patients treated with the intensive chemotherapy, a standard therapy for BL, MUM1-positive cases showed worse prognosis. Although the authors suggest that these two groups need separation, the data are not strong enough yet for such a conclusion.

## Ancillary techniques

Klapper et al. [37] described earlier in this Journal the importance of a good protocol for the assessment of proliferation by Ki 67 in MCL. Now, Scott et al. [38] describe a new molecular assay for the proliferation signature usable in formalin-fixed paraffin-embedded material (FFPE), the MCL35 assay, which contains a 17-gene signature. Forty-seven FFPE biopsies were used to train the assay on the NanoString platform, using microarray gene expression data of matched fresh frozen biopsies as a gold standard. Then, the assay was applied to pretreatment FFPE lymph node biopsies from an independent cohort of 110 uniformly treated patients. Seventeen biopsies were tested across three laboratories to assess assay reproducibility. The yielded gene expression was of sufficient quality to assign an assay score and risk group in 108 (98%) of the 110. The MCL35 assay assigned patients to high-risk (26%), standard-risk (29%), and low-risk (45%) groups, with different lengths of overall survival: a median of 1.1, 2.6, and 8.6 years, respectively. Concordance of risk assignment across the three independent laboratories was 100%. The MCL35 assay for FFPE biopsies therefore seems a reliable biomarker, but correlation with the extensively used Ki67 index is not yet done.

Rossi et al. [39] bark on the increasingly popular liquid biopsy, which is actually a hype word for blood test. Nevertheless, the possibility of deriving genetic data from tumors using a blood sample has great potential. Using ultra-deep targeted next-generation sequencing of pretreatment plasma cell-free (cf)DNA from DLBCL patients correctly discovered DLBCL-associated mutations that were represented in >20% of the alleles of the tumor biopsy with >90% sensitivity and ∼100% specificity. Plasma cfDNA genotyping also allowed for the recovery of mutations that were undetectable in the tissue biopsy, conceivably because, due to spatial tumor heterogeneity, they were restricted to clones that were anatomically distant from the biopsy site. Longitudinal analysis of plasma samples collected under rituximab-cyclophosphamide-doxorubicin-vincristine-prednisone (R-CHOP) chemotherapy showed a rapid clearance of DLBCL mutations from cfDNA among responding patients. Conversely, among patients who were resistant to R-CHOP, basal DLBCL mutations did not disappear from cfDNA. In addition, among treatment-resistant patients, new mutations were acquired in cfDNA that marked resistant clones selected during the clonal evolution. These results demonstrate that cfDNA genotyping of DLBCL is as accurate as genotyping of the diagnostic biopsy to detect clonally represented somatic tumor mutations and is a real-time and noninvasive approach to tracking clonal evolution and the emergence of treatment-resistant clones. When confirmed, this approach may radically alter hematopathology practice.
